# Invasive type B2 thymoma associated with myasthenia gravis, resection with superior vena cava reconstruction

**DOI:** 10.1093/jscr/rjac368

**Published:** 2022-09-05

**Authors:** Sawley A Wilde, William P Carroway, Diana S Hsu, Kian C Banks, Jeffrey B Velotta

**Affiliations:** Department of Surgery, UCSF East Bay, Oakland, CA, USA; Department of Surgery, UCSF East Bay, Oakland, CA, USA; Department of Surgery, UCSF East Bay, Oakland, CA, USA; Department of Surgery, UCSF East Bay, Oakland, CA, USA; Department of Thoracic Surgery, Kaiser Oakland Medical Center, Oakland, CA, USA

## Abstract

After being diagnosed with myasthenia gravis, a 55-year-old male was referred for treatment of an invasive thymoma. Preoperative imaging revealed a thymoma adjacent to the superior vena cava (SVC) with possible invasion of the left innominate vein. After multidisciplinary discussion, he underwent upfront en bloc resection of the tumor with SVC resection and reconstruction. He was discharged after an uncomplicated postoperative course with improvement of his symptoms.

## INTRODUCTION

The association between myasthenia gravis (MG) and thymomas is well established, as $\sim$30–40% of cortical thymoma patients develop MG, whereas 15% of MG patients have thymomas [1]. These thymomas are typically small and early stage tumors that are often discovered by screening chest computed tomography (CT). We present the case of a 55-year-old male who was first diagnosed with MG, then found to have a thymoma with imaging concerning for tumor compression of the superior vena cava (SVC) and possible left innominate vein involvement. He underwent upfront resection through a sternotomy incision, as well as SVC resection and reconstruction while on cardiopulmonary bypass (CPB).

## CASE REPORT

**Figure 1:**
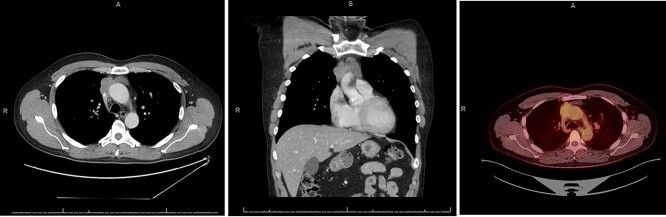
CT chest axial (left), coronal (middle) and PET (right) of invasive thymoma.

A 55-year-old male initially presented to his primary care provider with diplopia and eyelid droop. He was diagnosed with MG via confirmation of positive acetylcholine receptor blocking antibodies. CT chest revealed a complex, multilobulated, 6 cm anterior mediastinal mass compressing the SVC and possibly involving the central aspect of the left innominate vein(Fig. 1). A core needle biopsy was obtained, and pathology was consistent with type B2 thymoma, with no evidence of carcinoma. A positron emission tomography (PET) scan was also obtained, which revealed a maximum standard uptake value (SUVmax) of 3.0. Treatment was discussed across multiple disciplines at multiple institutions, with differing opinions regarding neoadjuvant chemotherapy, definitive chemoradiation due to unresectability, versus upfront surgical resection. Our facility, a major regional thoracic oncology center, was consulted and recommended upfront surgical resection followed by likely adjuvant radiation.

Before resection, he underwent MG optimization with a 3-day treatment course with IVIG and was asymptomatic on the day of operation. After standard median sternotomy incision, the tumor was identified and noted to be firm, adherent to the sternal wall and right upper lobe of the lung. In addition, it wrapped 270° around the SVC and had invaded and obliterated the lumen of the left innominate vein. A Vein of Marshall, identified preoperatively on CT scan, was also identified at this time and appeared free from involvement and was providing outflow from the left upper extremity, thus this was left patent to allow for appropriate collateral circulation.

**Figure 2:**
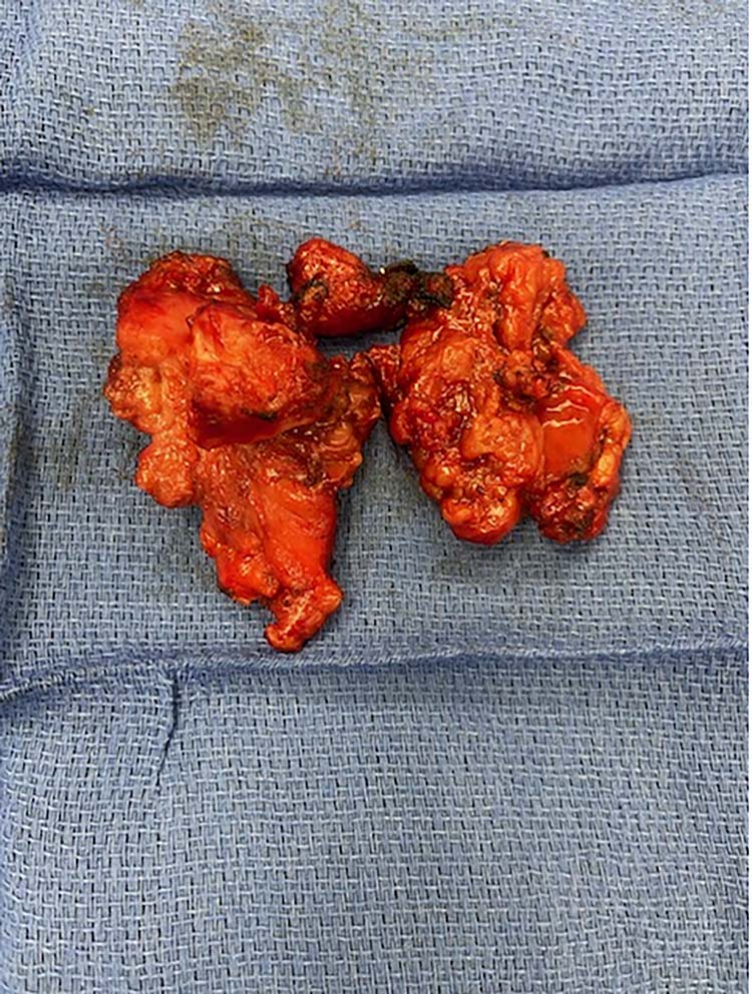
Post resection thymoma with en bloc resection of SVC.

**Figure 3:**
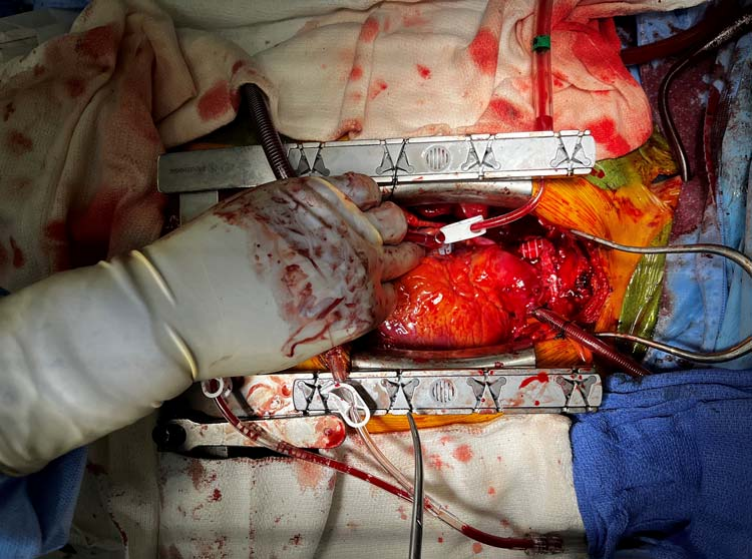
SVC with Gore-Tex graft reconstruction.

The decision was made to place the patient on CPB when it was evident the tumor would not safely dissect off the SVC. Then, careful dissection was performed around the SVC and innominate vein. The SVC was resected proximally and distally to the tumor, as well as en bloc wedges resection of the right upper lobe of the lung (Fig. 2). Frozen sections confirmed negative margins, and the SVC was reconstructed using 10 mm Gore-Tex tube graft, with good flow confirmed with intraoperative Doppler. The azygous vein was kept open and drained into the SVC, and the Vein of Marshall was left patent and continued to provide outflow after ligation of the already occluded innominate vein (Fig. 3).

Postoperatively, the patient’s course was uncomplicated. Chest tubes were removed on postoperative day (POD) 2, epicardial pacing wires on POD 3 and he was discharged home on aspirin on POD 5. Final pathology confirmed Stage III thymoma, type B2 with one positive microscopic margin on the posterior SVC. Due to the R1 resection, the patient underwent adjuvant 60Gy radiation to the mediastinum and is recovering well. He currently remains with no evidence of disease recurrence.

## DISCUSSION

Once the diagnosis of MG has been established, it is common practice to obtain chest CT to screen for thymoma, given the increased incidence [1]. When found, these tumors are often small, early stage and have no invasion. Surgical resection is the treatment of choice, with these resections frequently performed using minimally invasive techniques [2]. In this case, our patient presented with a larger tumor and with possible invasion into the SVC and innominate vein, despite having no compressive symptoms. There are limited data and controversy exists regarding first-line treatment of invasive thymoma, specifically in the setting of MG. Alternatives to surgery such as induction chemotherapy or definitive chemoradiation are often discussed [3]. This allows for downstaging of the tumor and may improve the likelihood that the tumor can be resected with negative microscopic margins. However, previous studies have demonstrated that when feasible, initial surgery provides better progression-free and overall survival [4], and chemotherapy regimens and response rates are highly variable [3, 5]. Thus, when amenable, surgery is still recommended as first line, even in cases as ours that may require more invasive techniques [1].

It is important to differentiate between invasive thymoma and thymic carcinoma, as the disease free and long-term survival are significantly different, and thus when feasible, biopsy is recommended [4]. In cases of thymic carcinoma, definitive chemoradiation is often the treatment of choice, given the much poorer prognosis [3]. In addition to CT, PET has also been described for determining invasiveness and prognosis. Tumors with a high SUVmax have an increased likelihood of carcinoma, with some studies reporting a value of 6 to predict carcinoma with high sensitivity [6]. Thymomas with higher SUVs have been thought to be associated with more invasive disease, although this has been shown to be much less predictable [6], as in this case, where the tumor had low SUV uptake despite having a high degree of invasion upon intraoperative assessment.

Intraoperatively, this case also presented many challenges that are not typically encountered in thymoma resections. Given the adherence to the great vessels, dissection could not be performed safely without the use of CPB and cardioplegia. The lumen of the left innominate vein was completely obliterated; however, the patient remained asymptomatic preoperatively as he had venous return through collateral vessels, including a patent Vein of Marshall, which is typically seen in <3% of the population [7]. Finally, obtaining a complete resection required resection and reconstruction of the SVC using a Gore-Tex graft on CPB.

This case demonstrates that although upfront surgical resection for stage III thymomas may involve more invasive techniques, when performed at high volume centers with a multidisciplinary team, it can safely be performed with successful outcomes and can still be considered as an optimal first line treatment.

## CONFLICT OF INTEREST STATEMENT

None declared.

## FUNDING

None.
